# Factor Structures in the Depressive Symptoms Domains in the 9Q for Northern Thai Adults and Their Association with Chronic Diseases

**DOI:** 10.3390/bs14070577

**Published:** 2024-07-07

**Authors:** Suttipong Kawilapat, Patrinee Traisathit, Narong Maneeton, Sukon Prasitwattanaseree, Thoranin Kongsuk, Suwanna Arunpongpaisal, Jintana Leejongpermpoon, Supattra Sukhawaha, Benchalak Maneeton

**Affiliations:** 1 Department of Statistics, Faculty of Science, Chiang Mai University, Chiang Mai 50200, Thailand; suttipong.kawilapat@cmu.ac.th (S.K.); patrinee.t@cmu.ac.th (P.T.); sukon.pra@cmu.ac.th (S.P.); 2 Department of Psychiatry, Faculty of Medicine, Chiang Mai University, Chiang Mai 50200, Thailand; narong.m@cmu.ac.th; 3 Prasrimahabhodi Psychiatric Hospital, Ubon Ratchathani 34000, Thailand; tkongsuk@gmail.com (T.K.); virgojinny12@gmail.com (J.L.); s_sukawa@yahoo.com (S.S.); 4 Somdet Chaopraya Institute of Psychiatry, Bangkok 10600, Thailand; 5 Department of Psychiatry, Faculty of Medicine, Khon Kaen University, Khon Kaen 40002, Thailand; suwaru@kku.ac.th

**Keywords:** depressive symptoms, cognitive-affective symptoms, somatic symptoms, Nine-Questions Depression-Rating Scale, Multiple Indicators Multiple Causes model

## Abstract

Most of the common models to examine depression are one-factor models; however, previous studies provided several-factor structure models on each depressive symptom using the Patient Health Questionnaire-9 (PHQ-9). The Nine-Questions Depression-Rating Scale (9Q) is an alternative assessment tool that was developed for assessing the severity of depressive symptoms in Thai adults. This study aimed to examine the factor structure of this tool based on the factor structure models for the PHQ-9 provided in previous studies using confirmatory factor analysis (CFA). We also examined the association of chronic diseases and depressive symptoms using the Multiple Indicators Multiple Causes model among 1346 participants aged 19 years old or more without psychiatric disorders. The results show that the two-factor CFA model with six items in the cognitive-affective domain and three items in the somatic domain provided the best fit for depressive symptoms in the study population (RMSEA = 0.077, CFI = 0.953, TLI = 0.936). Dyslipidemia was positively associated with both cognitive-affective symptoms (*β* = 0.120) and somatic depressive symptoms (*β* = 0.080). Allergies were associated with a higher level of cognitive-affective depressive symptoms (*β* = 0.087), while migraine (*β* = 0.114) and peptic ulcer disease (*β* = 0.062) were associated with a higher level of somatic symptoms. Increased age was associated with a lower level of somatic symptoms (*β* = −0.088). Our findings suggested that considering depressive symptoms as two dimensions yields a better fit for depressive symptoms. The co-occurrence of chronic diseases associated with depressive symptoms should be monitored.

## 1. Introduction

Depressive disorders, among the most common mental disorders, are a leading cause of the global disease burden and deaths by suicide. It has been estimated that 264 million people worldwide (3.44%; range 2–6%) and 2.62 million people in Thailand (3.09%) have experienced depression [1]. The symptomatology of depression includes somatic components (e.g., fatigue, poor appetite, concentration difficulty, bodily discomfort, poor memory, and loss of sexual drive) [2] and cognitive and affective components (e.g., sadness, negative thoughts, anhedonia, indecisiveness, and suicidal ideation) [3]. Some symptoms of depression, especially somatic symptoms such as fatigue, sleep problems, appetite changes, or psychomotor retardation, overlap with other disorders [4,5,6,7].

Socio-demographic factors (e.g., sex, age, marital status, income, and educational level) have been associated with depressive symptoms [8,9,10]. The outcomes from a previous longitudinal study involving cohorts of Canadians, Americans, and Europeans revealed an association between depressive symptoms and age, which was more prevalent in females than males [11]. It has also been reported that Korean individuals in the middle- and lower-income brackets have a higher likelihood of depressive symptoms [12].

The association between chronic disease and depression has been extensively explored [13,14,15,16,17,18]. In a recent study of older adults in Thailand with non-communicable diseases [18], the authors found that diabetes mellitus was associated with a higher risk of depression. In addition, depressive symptoms have also been associated with mortality and worsening health conditions. In a study of patients with chronic heart disease [19], the authors reported that patients with high scores for somatic/affective depressive symptoms had a higher incidence of mortality. Allergies have also been reported to be associated with a higher risk of depression and depressive symptoms [20,21,22,23]. In a recent cohort study involving breast cancer patients in France [24], the authors found that the patients had a higher probability of experiencing subthreshold or clinically significant depressive symptoms between diagnosis and after the end of breast cancer treatment. The results of a previously conducted systematic review and meta-analysis revealed that migraine can increase the incidence of depression [25]. The outcomes form a prospective study of patients with migraine also indicated that patients scored higher on acute depressive symptoms on the day of the migraine headache than on all other days during the migraine attack [26]. The author of another cohort study also found that migraine at baseline is associated with a higher risk of first-onset major depression while major depression at baseline is associated with a higher risk of migraine during follow-up [27]. A bidirectional association between chronic kidney disease (CKD) and depressive symptoms was discovered among patients in China [28]: depression was significantly positively associated with CKD while CKD diagnosis was also significantly associated with an increased risk of incident depression compared to the non-CKD group. The results from a network analysis to examine the longitudinal associations of depressive symptoms among patients with CKD before and after diagnosis of CKD indicate that fatigue and less happiness before diagnosis of CKD were associated with other symptoms at the time of diagnosis, while a depressed mood at the time of diagnosis was associated with other symptoms in postdiagnosis [29].

We previously conducted a study to develop an Item Response Theory (IRT)-based weighed-scoring approach for the Nine-Question Depression-Rating Scale (9Q) using a standard one-factor model [30], which improved the precision of the scoring method compared to the traditional sum-score approach. Nevertheless, the fit indices for the model intimated that model fitting could be improved. Thus, we hypothesized that a two-factor model might improve the fit of the model for the 9Q and help to identify a more precise scoring method. Previously, researchers have explored the structure of depressive symptoms and measurement tools for depressive symptoms using a standard one-factor model [31,32,33,34,35,36] or a two-factor model [37,38,39,40,41,42]. However, the components of the factors varied in each study. Therefore, to find the model providing the best fit for the assessment of depressive symptoms, our aim was to explore the factor structure of the 9Q with a two-factor model and compare its performance with other models proposed in previous studies.

We previously discovered differential item functioning in some items of the 9Q across sex [30], which might account for differences between the responses of males and females for some of the depressive symptoms. In the present study, we also explored the association between socio-demographic factors and chronic diseases and depressive symptoms adjusted for sex. Thus, we used a Multiple Indicators, Multiple Causes (MIMIC) model to detect potential differential item functioning and examined the association between exogenous covariates and latent variables.

## 2. Materials and Methods

### 2.1. Participants and Study Design

**Figure 1:**
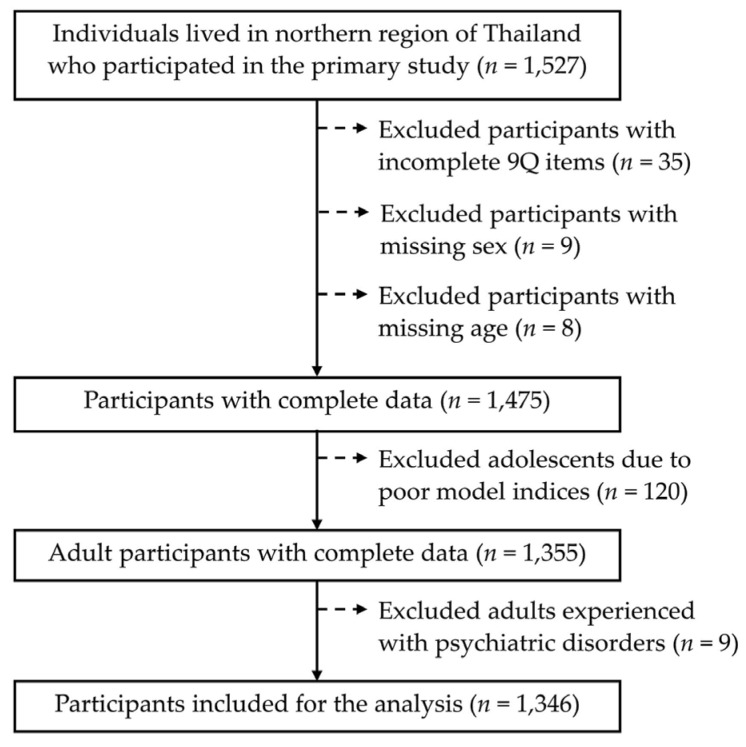
A flow chart of the study population inclusion process.

For this psychometric study, we used secondary data from a cross-sectional study on the criterion-related validity of the revised 9Q in the northern Thai dialect. Of the 1527 participants in the primary study, 181 were excluded due to incompleteness of the 9Q items endorsement (*n* = 35), missing sex (*n* = 9), missing age (*n* = 8), age < 18 years old (*n* = 120), and having been previously diagnosed with a psychiatric disorder (*n* = 9). Adolescents were excluded due to the poor confirmatory factor analysis indices when included, while the adults who had previously been diagnosed with a psychiatric disorder were excluded to avoid confounding with other diseases in the association analysis (Figure 1). Therefore, 1346 participants who completed all of the items in the assessment, were aged ≥ 19 years old, and had not previously been diagnosed with a psychiatric disorder were included.

The sample size required for the primary study according to the sample size calculation for a diagnostic test (a sensitivity of 0.90 with 10% precision, a 95% confidence interval, and the prevalence of depression in the northern region of Thailand being 2.3%) was 1505 individuals [43]. The required sample size was considered via a rule-of-thumb approach for latent variable models with continuous outcomes using maximum likelihood estimation (i.e., 10–20 individuals per parameter) [44]. Since we considered 59 parameters for the MIMIC model (19 parameters for exogenous variables to each latent variable, 9 for latent variables to the observed variables, 1 covariance parameter between latent variables, and 11 error parameters), the minimum required sample size should be 590–1180 individuals. The participants were selected via systematic random sampling of the alphabetic order of the names on the house registration record.

### 2.2. Variables and Measurements

#### 2.2.1. Instrument

The instrument used in this study was a revised 9Q translated from the central Thai dialect version (Cronbach’s alpha = 0.821) [45] to the northern Thai dialect. The latter yielded adequate construct validity (comparative fit index (CFI) = 0.949, Tucker–Lewis index (TLI) = 0.932, and root mean squared error of approximation (RMSEA) = 0084) and good internal consistency (Cronbach’s alpha = 0.855) [30]. The 9Q was developed to assess the severity of depressive symptoms, which accounted for both the frequency and symptom intensity within the previous two weeks. The 9Q consists of nine rating scale items: mood, anhedonia, sleep disturbance, fatigue, appetite changes, guilt, concentration problems, psychomotor retardation, and suicidal ideation. The total score of the 9Q is the product of the intensity (0, 1, 2, and 3 points for no, mild, moderate, and severe symptoms, respectively) and frequency (1, 2, and 3 points for several days, more than a week, and nearly every day, respectively) of each item, which ranges from 0 to 81 points.

#### 2.2.2. Study Variables

The covariates examined in this study included both socio-demographics factors (sex, age, relationship status, educational level, occupation, and income) and chronic diseases (cancer, chronic kidney disease (CKD), coronary artery disease (CAD), asthma, diabetes mellitus (DM), hypertension, dyslipidemia (DLP), migraine, peptic ulcer disease (PUD), thalassemia, thyroid, rheumatism/gout, and allergies). Any other diseases with less than 10 patients were as assigned to “other diseases”.

### 2.3. Prior Models of Depressive Smptoms Structure

**Figure 2:**
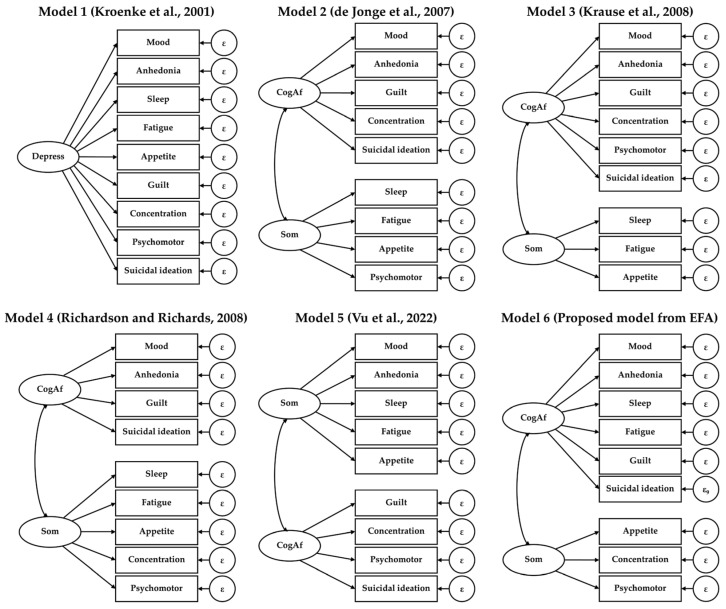
Confirmatory factor analysis models for examining factor structures of the Nine-Questions Depression-Rating Scale (9Q). CogAf, cognitive-affective symptoms; Som, somatic symptoms [31,37,40,41,42].

Several previous studies were conducted and suggested different model structures for depressive symptoms, including both one- and two-factor models based on the Patient Health Questionnaire-9 (PHQ-9). Since the 9Q was recently developed, we considered comparing our proposed structure model from the exploratory factor analysis (EFA) with five prior models with different components suggested in the previous studies [31,37,40,41,42] (Figure 2).

### 2.4. Statistical Analysis

We conducted an EFA of the 9Q for an appropriate two-factor model regarding the observed data. Items with a factor loading value ≥ 0.35 were considered as components in the relevant domain [46]. Cronbach’s alpha coefficients were used to measure the internal consistency of each domain. The alpha coefficient ≥ 0.70 was considered acceptable [47].

Confirmatory factor analysis (CFA) was applied to examine the fit of model structure. The relatively good fit indices of the CFA model were considered from a CFI > 0.95, a TLI > 0.95, and a RMSEA < 0.06 [48]. Hooper D et al. [49] also suggested that CFI > 0.90, TLI > 0.90, and RMSEA < 0.08 indicated an acceptable fit. The model with the lowest score of Akaike information criterion (AIC) and Bayesian information criterion (BIC) would also be considered.

A MIMIC model based on maximum likelihood with missing values estimation was applied to explore the association between the chronic diseases after evaluating the measurement model. The exogenous covariates included in the MIMIC model include sex, age, divorce/widowed, income, educational level, employment, and chronic diseases.

A two-sided *p*-value of <0.05 was considered statistically significant. All analyses were performed using Stata 17.0 (StataCorp LP, College Station, TX, USA).

## 3. Results

### 3.1. Characteristics and Depressive Symptoms of the Participants

Of the 1346 participants aged 19 years old or more, the majority were female (68.05%) and adults aged 19–59 years (80.16%). Most participants were married or living with a partner (65.13%). Only two-fifths were divorced or widowed (17.17%). One-third of the participants reported having chronic diseases (37.32%). The most prevalent disease was hypertension (19.08%), followed by DM (8.19%) and DLP (6.01%) (Table 1). 

Females reported a higher prevalence of anhedonia than males (25.87% vs. 17.91%). Adults aged 60 years old or more had a lower prevalence of anhedonia (17.98% vs. 24.65%) but a higher one for fatigue (16.48% vs. 10.84%) compared to younger adults. The participants who had not received an education reported a high prevalence of several depressive symptoms, including mood (37.50%), anhedonia (37.50%), sleep disturbance (29.17%), fatigue (41.67%), appetite change (33.33%), concentration problems (29.17%), psychomotor retardation (33.33%), and suicidal ideation (12.50%). The participants with chronic diseases reported a higher prevalence of sleep disturbance (31.67% vs. 19.30%), fatigue (16.33% vs. 9.41%), appetite changes (20.92% vs. 14.23%), and psychomotor retardation (23.90% vs. 13.03%) than those without them. A high prevalence of mood symptoms was reported by those with CAD (28.57%) and CKD (27.27%). The participants with cancer reported the highest prevalence of anhedonia (50%). The highest prevalence of sleep disturbance was reported by those with migraine (50%) followed by DLP (40%), thyroid (40%), CKD (36.36%), PUD (35.29%), and allergies (32.14%). Guilt was most predominant in those with CKD (36.36%), followed by asthma (34.78%) and cancer (30%). Psychomotor retardation was most commonly reported by the participants with allergies (42.86%), followed by migraine (40%), CKD (36.36%), CAD (35.71%), and PUD (35.29%). The highest prevalence of suicidal ideation was found in those with cancer (20%), followed by migraine (20%) and CAD (14.29%). The participants with CKD also reported the highest prevalence of fatigue (45.45%), followed by appetite change (45.45%) and concentration problems (36.36%) (Table S1).

### 3.2. Structure of Depressive Symptoms Measurement

A two-factor model, separated as cognitive-affective dimension (i.e., mood, anhedonia, sleep, fatigue, guilt, and suicide) and somatic dimension (i.e., appetite, concentration, and psychomotor), was identified using the EFA (Table 2), with an acceptable Cronbach’s alpha value of 0.816 for the cognitive-affective domain and 0.645 (close to the acceptance value) for the somatic domain. 

The CFA results indicate that Model 6 with two factors provided better fit index values than the previous two-factor models (Models 2–5) and the traditional one-factor model (Model 1) (RMSEA = 0.077, CFI = 0.953, TLI = 0.936, AIC = 31,280.514, and BIC = 31,426.251). Factor correlation between the cognitive-affective and somatic dimensions was also the lowest (0.771) (Table 3).

### 3.3. Associations with Chronic Diseases and Covariates

**Figure 3:**
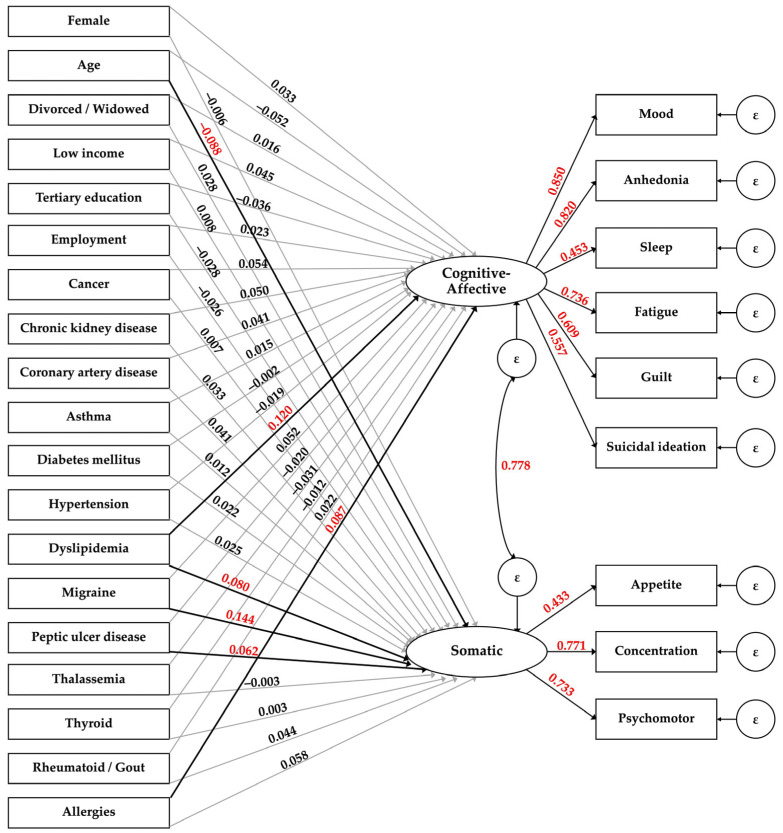
Multiple Indicators Multiple Causes (MIMIC) model of the Nine-Questions Depression-Rating Scale (9Q) for cognitive-affective and somatic depressive symptoms among northern Thai adults. The black lines represent the paths with significant standardized coefficients which are marked in red color.

After adding the covariates to Model 6, the model fit indices declined but remained within the acceptable ranges (RMSEA = 0.040, CFI = 0.927, TLI = 0.905), and factor loadings remained significant. The standardized coefficients of potential associated factors to each domain of depressive symptoms are shown in the MIMIC model (Figure 3). 

Exposure to DLP was positively associated with both cognitive-affective symptoms (*β* = 0.120; *p* < 0.001) and somatic depressive symptoms (*β* = 0.080; *p* = 0.019). Allergy exposure was associated with a higher level of cognitive-affective depressive symptoms (*β* = 0.087; *p* = 0.002), while having a migraine (*β* = 0.114; *p* < 0.001) or PUD (*β* = 0.062; *p* = 0.048) was associated with a higher level of somatic symptoms. Increased age was associated with a lower level of somatic symptoms (*β* = −0.088; *p* = 0.025). Other socio-demographic covariates of participants were not associated with both dimensions of depressive symptoms (Table 4).

## 4. Discussion

We found that a two-factor model with cognitive-affective and somatic domains could better describe the symptoms of depression than a previously reported one-factor model. This finding is consistent with several other studies in which a two-factor model was used, albeit the components in each domain were different. Sleep and fatigue components were considered cognitive-affective symptoms, which is inconsistent with their previous inclusion in the somatic domain [37,40,41,42]. According to the factor loadings in the EFA results, fatigue had quite similar loadings in both domains. We hypothesized that fatigue and sleep could overlap between the two domains especially for participants who had comorbidity with other psychiatric disorders. The authors of a previous study of Australian adults found that fatigue and sleep disturbance are independently associated with many psychiatric disorders [50].

The results from a previous longitudinal study in China revealed that chronic diseases are linked with a significantly higher risk of depression that varies according to the specific diseases [51]; the authors also found that the risk of depression increased with the number of chronic diseases. The outcomes from a previous study on the US populations revealed that having a higher number of chronic diseases increases the likelihood of fatigue, psychomotor retardation, and sleep disturbance [52]. Although we did not examine the association of chronic diseases with specific depressive symptoms, we also found significant associations between some chronic diseases and the cognitive-affective or somatic domains. 

DLP, with a higher level of depressive symptoms than the other diseases, was the only disease associated with both the cognitive-affective and somatic domains in our study. The outcomes from several previous studies in various settings consistently indicate that DLP and high cholesterol are associated with depressive symptoms [53,54,55,56,57,58]. In a recent study of the plasma lipidomic profile and depressive symptoms among Native Americans [59], the authors suggested that changes in sphingomyelins, glycerophospholipids, acylcarnitines, fatty acids, and triacylglycerols are linked to changes in depressive symptoms. The results from a study of older people in China [57] also suggest mediating effects of ischemic heart disease and stroke on the associations of serum lipids with depressive symptoms. However, we were unable to be that specific due to the small number of participants with these diseases in our study.

Allergy was found to be associated with a higher level of cognitive-affective depressive symptoms in our study. This is consistent with the outcomes from previous studies inferring that allergic disorders are significantly associated with an increased risk of depression [20,22]. In a study of seasonal allergic rhinitis patients [21], the authors found a significant increase in depression self-report scores during acute allergic inflammation episodes compared to the asymptomatic period and the non-allergic group; they suggested that the inflammatory markers for the allergy were potentially associated with an increase in moodiness and sleep quality deprivation during the acute allergic period.

Several studies have previously been conducted to explore the risk of PUD occurrence among patients with depression [60,61,62] rather than the risk of depression among PUD patients. The authors found that patients with depression had a higher risk of developing PUD. Interestingly, our study also revealed an association between PUD and depressive symptoms. We found that participants with PUD had a higher level of somatic depressive symptoms than those without PUD. However, the cross-sectional nature of our study means that we could not distinguish between the cause and the effect. Nevertheless, in a recent study in Taiwan on UD patients undergoing antibiotic treatment for *Helicobacter pylori* infection [63], the authors found that patients receiving antibiotic therapy had an increased risk of short-term depressive disorder. Therefore, monitoring the effects of the treatment for PUD and other associated chronic diseases should be conducted by physicians or other healthcare providers.

Breslau et al. [27] discovered a bidirectional association between migraine and major depression; they found that migraine at baseline is associated with a higher risk of first-onset major depression, while major depression at baseline is associated with a higher risk of migraine during follow-up. In a study of the general Brazilian population [64], the authors reported that all of the PHQ-9 items were significantly related to migraine compared to the control group. Moreover, the physical symptoms of depression (appetite, fatigue, and poor sleep) had stronger links to migraine than the emotional symptoms (hopelessness and sadness). Jahangir et al. [65] provided a detailed review of the molecular genetic background of the relationship between migraine and depression. Our findings also confirmed an association between migraine and somatic symptoms. In addition to sleep disturbance, 40% of the participants with migraine reported a high proportion of psychomotor retardation. However, since the number of participants with migraine reported in our study was low, this finding should be confirmed by conducting a further study with a larger sample size.

In a previous cohort study of women in France [24], the authors found that patients with breast cancer experienced temporally or lasting significant depressive symptoms during and after treatment; the presence of depressive symptoms could be due to both demographic (low household income and a family history of breast cancer) and clinical (cancer stage, a high level of fatigue, and depression at diagnosis) factors. The outcomes from a recent study of cognitive variables on patients with cancer over five years of follow-up suggest that rumination is significantly associated with depressive symptoms [66]. Similarly, CKD has been reported to be associated with an increased risk of depression [28], albeit the association was bidirectional. However, neither cancer nor CKD was significantly associated with depressive symptoms in our study, although there was some evidence of an association in the cognitive-affective domain. These results are likely to the very low number of participants with each chronic disease. Further studies with a larger number of participants with these diseases should be conducted to explore this phenomenon.

The associations between depressive symptoms and some chronic diseases (such as migraine, DLP, PUD, and allergy) suggest that individuals with the co-occurrence of these diseases could cause higher levels of depressive symptoms in sufferers than those without the diseases. In addition, some depressive symptoms that are associated with chronic diseases could cause other symptoms. In a previous study in China [29], the authors conducted a network analysis to examine the longitudinal associations of depressive symptoms from before and after a diagnosis of CKD; they found that fatigue and less happiness before diagnosis of CKD were associated with other symptoms at diagnosis, while depressed mood at the time of diagnosis was associated with other symptoms during the postdiagnosis period. They suggested identifying symptoms early and appropriately managing them could be beneficial in reducing the risk of activating other depressive symptoms. However, since we used cross-sectional data, we were unable to examine the sequences of depressive symptoms. A longitudinal study to uncover any potential relationships between depressive symptoms with other chronic diseases could be advantageous. 

We also found an association between age and somatic symptoms, with older participants having a less of them. This is consistent with the findings from a previous longitudinal study of Canadian, American, and European cohorts [11], in which the authors found a negative association between depressive symptoms and age during middle age but a positive one during later life. They also found that females reported greater depressive symptoms than males and discovered an interaction between sex and age in some of the cohorts; this could be an important mediator for the different outcomes for middle age and later life. 

Sex has also been reported to be associated with both the cognitive-affective and somatic domains in a study of patients with heart disease in Brazil [9], which contrasts with our findings. Even though we did not find an association between sex and depressive symptoms, the outcomes from our previous study point toward differential item functioning in some items of the 9Q [30], which could account for the difference between the responses for some of the somatic symptoms between males and females. Likewise, it might also be differential item functioning in other covariates. Identifying the differential item functioning of an item across population groups might make the scoring method more precise. Therefore, the association between covariates and specific depressive symptoms should be studied further.

In a previous study in the US [52], the authors reported that social risk factors (e.g., divorce, poverty, and lack of support) were associated with most cognitive-affective symptoms except for concentration problems. Inconsistently, we did not find any association between divorce/widowhood and poverty in either domain of depressive symptoms, which could be due to differences in the cultural and social contexts between the studies. 

The strengths of our study are the varied study settings across the northern region of Thailand and the exploration of chronic diseases adjusted using socio-demographic covariates to uncover their potential association with depressive symptoms. However, there are also some limitations. First, there was only a small number of participants with some of the included chronic diseases: cancer, CKD, CAD, migraine, PUD, and thalassemia. Thus, generalization should be approached with some caution because it would lower the statistical power of the model. Second, the number of female participants was much higher than the males (68% vs. 32%), which could have affected the outcome, even though sex was adjusted for in the model. Third, some characteristics that are potentially associated with depressive symptoms, such as social support, quality of life, length of widowhood, chronic disease duration, anxiety, stress, and other psychiatric conditions that may overlap with depression were not included in this study. Thus, a further study that includes these as covariates should be conducted. Finally, we only compared one- and two-factor models; other factor structure models (e.g., high-order and bi-factor) could have also been considered.

## 5. Conclusions

We found that considering depressive symptoms in two dimensions (the cognitive-affective and somatic domains) yielded a better model fit for depressive symptoms. This suggests the benefits of using a multidimensional item response theory model for more precise scoring of the 9Q. Some chronic diseases were positively associated with specific depressive symptom domain (DLP and allergies with the cognitive-affective domain and DLP, migraine, and PUD with the somatic domain). Moreover, depression associated with the co-occurrence of these chronic diseases should be monitored. We also found a negative association between age and somatic symptoms. A future study in which the comorbidity of psychiatric conditions and other poignant covariates, along with other factor structure models, should be conducted to further investigate these findings. In addition, differential item functioning across sex or chronic diseases should be examined.

## Figures and Tables

**Table 1:** Characteristics of the participants (*n* = 1346).

Covariates	*n* (%)
Sex	
Male	430 (31.95%)
Female	916 (68.05%)
Age (mean = 47.0, SD = 14.5)	
19–59 years old	1079 (80.16%)
≥60 years old	267 (19.84%)
Relationship status (*n* = 1345)	
Single	238 (17.70%)
Married/with a partner	876 (65.13%)
Divorced	99 (7.36%)
Widowed	132 (9.81%)
Educational level (*n* = 1336)	
None	24 (1.80%)
Primary school	609 (45.58%)
Lower secondary school	195 (14.60%)
Upper secondary school	182 (13.62%)
Diploma	171 (12.80%)
Bachelor	139 (10.40%)
Masters	16 (1.20%)
Occupation (*n* = 1339)	
Employee	598 (44.66%)
Government official	78 (5.83%)
Merchant	148 (11.05%)
Agriculturist	220 (16.43%)
Business owner	52 (3.88%)
Student	32 (2.39%)
Unemployed	211 (15.76%)
Income (USD/month) ^a^ (*n* = 1331)	
0–150	647 (48.61%)
151–300	433 (32.53%)
301–600	198 (14.88%)
601–1200	39 (2.93%)
>1200	14 (1.06%)
Chronic diseases ^b^ (*n* = 1331)	
No	829 (62.28%)
Yes	502 (37.72%)
Cancer	10 (0.75%)
Chronic kidney disease	11 (0.83%)
Coronary artery disease	14 (1.05%)
Asthma	23 (1.73%)
Diabetes mellitus	109 (8.19%)
Hypertension	254 (19.08%)
Dyslipidemia	80 (6.01%)
Migraine	10 (0.75%)
Peptic ulcer disease	17 (1.28%)
Thalassemia	13 (0.98%)
Thyroid	30 (2.25%)
Rheumatoid/gout	25 (1.88%)
Allergies	28 (2.10%)
Other diseases	62 (4.66%)

*n*, number of available observations; SD, standard deviation. ^a^ 1 USD is approximately 33 Thai baht. ^b^ Some participants had multiple diseases.

**Table 2:** Exploratory factor analysis of the Nine-Questions Depression-Rating Scale (9Q) for northern Thai adults (*n* = 1346).

Items	Factor 1: Cognitive/Affective	Factor 2: Somatic
1. Mood	0.799 *	0.294
2. Anhedonia	0.821 *	0.205
3. Sleep	0.350 *	0.327
4. Fatigue	0.690 *	0.271
5. Appetite	0.278	0.331 *
6. Guilt	0.478 *	0.398
7. Concentration	0.377	0.647 *
8. Psychomotor	0.305	0.688 *
9. Suicidal ideation	0.436 *	0.364
Cronbach’s alpha	0.798	0.645

* Factor loading ≥ 0.350.

**Table 3:** Factor loadings and correlation, and fitting-indices values for the studied models (*n* = 1346).

9Q Items	Model 1 ^a^	Model 2 ^b^	Model 3 ^c^	Model 4 ^d^	Model 5 ^e^	Model 6 ^f^
Factor loadings						
1. Mood	0.837	0.843	0.841	0.860	0.854	0.852
2. Anhedonia	0.803	0.806	0.805	0.816	0.830	0.820
3. Sleep	0.463	0.474	0.484	0.487	0.452	0.452
4. Fatigue	0.730	0.743	0.767	0.732	0.738	0.735
5. Appetite	0.403	0.413	0.420	0.429	0.391	0.429
6. Guilt	0.625	0.614	0.615	0.608	0.650	0.607
7. Concentration	0.639	0.636	0.639	0.674	0.721	0.783
8. Psychomotor	0.591	0.596	0.590	0.628	0.686	0.724
9. Suicide	0.565	0.565	0.565	0.562	0.584	0.557
Factor correlation	NA	0.972	0.941	0.917	0.833	0.771
Model fit						
RMSEA	0.104	0.105	0.104	0.100	0.082	0.077
CFI	0.913	0.913	0.915	0.922	0.948	0.953
TLI	0.884	0.880	0.882	0.893	0.928	0.936
SRMR	0.049	0.049	0.049	0.046	0.043	0.043
AIC	31,461.346	31,459.532	31,453.617	31,419.240	31,306.054	31,280.514
BIC	31,601.878	31,605.269	31,599.354	31,564.977	31,451.791	31,426.251

AIC, Akaike information criterion; BIC, Bayesian information criterion, RMSEA, root mean squared error of approximation; CFI, comparative fit index; TLI, Tucker–Lewis index; SRMR, standardized root mean squared residual; NA, not applicable. ^a^ One-factor model [31]. ^b^ Two-factor model [40]. ^c^ Two-factor model [41]. ^d^ Two-factor model [42]. ^e^ Two-factor model [37]. ^f^ Two-factor model from the exploratory factor analysis of the 9Q for northern Thai adults.

**Table 4:** Association of socio-demographics factors and chronic diseases with the cognitive-affective and somatic depressive domains (*n* = 1346).

Covariates	MIMIC Model		
	*β*	(95% CI)	*p*-Value
Cognitive-affective domain			
Female (ref: male)	0.033	(−0.025, 0.091)	0.267
Age (years old)	−0.052	(−0.123, 0.018)	0.148
Divorced/widowed (ref: single/married)	0.016	(−0.043, 0.075)	0.587
Income ≤ 300 USD (ref: >300 USD)	0.045	(−0.018, 0.108)	0.164
Diploma/bachelor’s/master’s degree (ref: high school or lower)	−0.036	(−0.104, 0.033)	0.306
Unemployed (ref: students/employed/agriculturist/business owner)	0.023	(−0.035, 0.081)	0.443
Cancer (ref: absence)	0.054	(−0.002, 0.110)	0.060
Chronic kidney disease (ref: absence)	0.050	(−0.007, 0.107)	0.086
Coronary artery disease (ref: absence)	0.041	(−0.016, 0.098)	0.156
Asthma (ref: absence)	0.015	(−0.041, 0.072)	0.597
Diabetes mellitus (ref: absence)	−0.002	(−0.064, 0.060)	0.954
Hypertension (ref: absence)	−0.019	(−0.085, 0.047)	0.569
Dyslipidemia (ref: absence)	0.120	(0.059, 0.181)	<0.001 *
Migraine (ref: absence)	0.052	(−0.005, 0.108)	0.072
Peptic ulcer disease (ref: absence)	−0.020	(−0.076, 0.037)	0.496
Thalassemia (ref: non-exposed)	−0.031	(−0.088, 0.025)	0.275
Thyroid (ref: absence)	−0.012	(−0.069, 0.045)	0.689
Rheumatoid/gout (ref: absence)	0.022	(−0.035, 0.079)	0.457
Allergies (ref: absence)	0.087	(0.031, 0.143)	0.002 *
Somatic domain			
Female (ref: male)	−0.006	(−0.069, 0.058)	0.861
Age (years old)	−0.088	(−0.165, −0.011)	0.025 *
Divorced/widowed (ref: single/married)	0.028	(−0.037, 0.092)	0.401
Income ≤ 300 USD (ref: >300 USD) ^a^	0.008	(−0.061, 0.077)	0.824
Diploma/bachelor’s/master’s degree (ref: high school or lower)	−0.028	(−0.102, 0.047)	0.466
Unemployed (ref: students/employed/agriculturist/business owner)	−0.026	(−0.089, 0.038)	0.426
Cancer (ref: absence)	0.007	(−0.055, 0.068)	0.836
Chronic kidney disease (ref: absence)	0.033	(−0.029, 0.095)	0.299
Coronary artery disease (ref: absence)	0.041	(−0.021, 0.103)	0.194
Asthma (ref: absence)	0.012	(−0.050, 0.073)	0.712
Diabetes mellitus (ref: absence)	0.022	(−0.045, 0.089)	0.521
Hypertension (ref: absence)	0.025	(−0.047, 0.097)	0.499
Dyslipidemia (ref: absence)	0.080	(0.013, 0.147)	0.019 *
Migraine (ref: absence)	0.144	(0.083, 0.205)	<0.001 *
Peptic ulcer disease (ref: absence)	0.062	(0.0004, 0.124)	0.048 *
Thalassemia (ref: non-exposed)	−0.003	(−0.064, 0.059)	0.932
Thyroid (ref: absence)	0.003	(−0.059, 0.064)	0.928
Rheumatoid/gout (ref: absence)	0.044	(−0.018, 0.106)	0.168
Allergies (ref: absence)	0.058	(−0.003, 0.120)	0.064

MIMIC, multiple indicators multiple causes; *β*, standardized coefficients; CI, confidence interval. ^a^ 1 USD is approximately 33 Thai baht. * Significant association (*p*-value < 0.05).

## Data Availability

The datasets used and/or analyzed during the current study are not publicly available due to lack of previous approval to share data publicly. The de-identified datasets used and/or analyzed during the current study can be made available through a data-sharing agreement with the corresponding author on reasonable request.

## Supplementary Materials

The following supporting information can be downloaded at https://www.mdpi.com/article/10.3390/bs14070577/s1. Table S1: Depressive symptoms of the participants (*n* = 1346).
