# Supplementary material for: Factor Structures in the Depressive Symptoms Domains in the 9Q for Northern Thai Adults and Their Association with Chronic Diseases
Source: Behav Sci (Basel). 2024 Jul 7;14(7):577. doi: 10.3390/bs14070577 (PMC11274117; doi:10.3390/bs14070577)
Supplement: Supplementary file 1 [file behavsci-14-00577-s001.zip › behavsci-2963406-supplementary.pdf]

Table S1. Depressive symptoms of the participants (*N* = 1,346).

Covariates	Total	Depressive Symptoms								
		Mood	Anhedonia	Sleep	Fatigue	Appetite	Guilt	Concentration	Psychomotor	Suicidal
Sex										
Male	430 (31.95%)	48 (11.16%)	77 (17.91%)	88 (20.47%)	46 (10.70%)	73 (16.98%)	53 (12.33%)	78 (18.14%)	75 (17.44%)	15 (3.49%)
Female	916 (68.05%)	138 (15.07%)	237 (25.87%)	234 (25.55%)	115 (12.55%)	153 (16.70%)	110 (12.01%)	182 (19.87%)	156 (17.03%)	39 (4.26%)
Age (Mean = 47.0, SD = 14.5)										
19–59 years old	1,079 (80.16%)	151 (13.99%)	266 (24.65%)	246 (22.80%)	117 (10.84%)	185 (17.15%)	133 (12.33%)	210 (19.46%)	180 (16.68%)	42 (3.89%)
≥ 60 years old	267 (19.84%)	35 (13.11%)	48 (17.98%)	76 (28.46%)	44 (16.48%)	41 (15.36%)	30 (11.24%)	50 (18.73%)	51 (19.10%)	12 4.49%)
Relationship status (*n* = 1,345)										
Single	238 (17.70%)	36 (15.13%)	76 (31.93%)	55 (23.11%)	21 (8.82%)	43 (18.07%)	40 (16.81%)	61 (25.63%)	43 (18.07%)	6 (2.52%)
Married/with a partner	876 (65.13%)	114 (13.01%)	185 (21.12%)	200 (22.83%)	112 (12.79%)	139 (15.87%)	94 (10.73%)	158 (18.04%)	146 (16.67%)	35 (4.00%)
Divorced	99 (7.36%)	15 (15.15%)	22 (22.22%)	28 (28.28%)	12 (12.12%)	20 (20.20%)	12 (12.12%)	16 (16.16%)	19 (19.19%)	5 (5.05%)
Widowed	132 (9.81%)	21 (15.91%)	31 (23.48%)	38 (28.79%)	16 (12.12%)	24 (18.18%)	17 (12.88%)	25 (18.94%)	23 (17.42%)	8 (6.06%)
Educational level (*n* = 1,336)										
None	24 (1.80%)	9 (37.50%)	9 (37.50%)	7 (29.17%)	10 (41.67%)	8 (33.33%)	3 (12.50%)	7 (29.17%)	8 (33.33%)	3 (12.50%)
Primary school	609 (45.58%)	93 (15.27%)	128 (21.02%)	186 (30.54%)	93 (15.27%)	112 (18.39%)	73 (11.99%)	110 (18.06%)	113 (18.56%)	33 (5.42%)
Lower secondary school	195 (14.60%)	31 (15.90%)	54 (27.69%)	35 (17.95%)	16 (8.21%)	42 (21.54%)	23 (11.79%)	40 (20.51%)	36 (18.46%)	6 (3.08%)
Upper secondary school	182 (13.62%)	24 (13.19%)	44 (24.18%)	33 (18.13%)	14 (7.69%)	19 (10.44%)	26 (14.29%)	33 (18.13%)	25 (13.74%)	4 (2.20%)
Diploma	171 (12.80%)	13 (7.60%)	40 (23.39%)	38 (22.22%)	13 (7.60%)	24 (14.04%)	19 (11.11%)	33 (19.30%)	24 (14.04%)	3 (1.75%)
Bachelor	139 (10.40%)	13 (9.35%)	34 (24.46%)	19 (13.67%)	11 (7.91%)	19 (13.67%)	18 (12.95%)	32 (23.02%)	20 (14.39%)	4 (2.88%)
Masters	16 (1.20%)	1 (6.25%)	4 (25.00%)	3 (18.75%)	2 (12.50%)	1 (6.25%)	0 (0%)	3 (18.75%)	2 (12.50%)	0 (0%)
Occupation (*n* = 1,339)										
Employee	598 (44.66%)	98 (16.39%)	161 (26.92%)	143 (23.91%)	72 (12.04%)	109 (18.23%)	85 (14.21%)	112 (18.73%)	112 (18.73%)	35 (5.85%)
Government official	78 (5.83%)	6 (7.69%)	15 (19.23%)	14 (17.95%)	6 (7.69%)	9 (11.54%)	7 (8.97%)	16 (20.51%)	11 (14.10%)	1 (1.28%)
Merchant	148 (11.05%)	21 (14.19%)	34 (22.97%)	31 (20.95%)	14 (9.46%)	21 (14.19%)	19 (12.84%)	31 (20.95%)	21 (14.19%)	2 (1.35%)
Agriculturist	220 (16.43%)	18 (8.18%)	34 (15.45%)	59 (26.82%)	28 (12.73%)	32 (14.55%)	14 (6.36%)	36 (16.36%)	36 (16.36%)	4 (1.82%)
Business owner	52 (3.88%)	3 (5.77%)	10 (19.23%)	6 (11.54%)	3 (5.77%)	8 (15.38%)	5 (9.62%)	5 (9.62%)	6 (11.54%)	1 (1.92%)
Student	32 (2.39%)	6 (18.75%)	11 (34.38%)	8 (25.00%)	0 (0%)	6 (18.75%)	7 (21.88%)	11 (34.38%)	4 (12.50%)	0 (0%)
Unemployed	211 (15.76%)	33 (15.64%)	47 (22.27%)	60 (28.44%)	37 (17.54%)	39 (18.48%)	25 (11.85%)	48 (22.75%)	40 (18.96%)	10 (4.74%)

Covariates	Total	Depressive Symptoms								
		Mood	Anhedonia	Sleep	Fatigue	Appetite	Guilt	Concentration	Psychomotor	Suicidal
Income (USD/month)[a] (*n* = 1,331)										
0–150	647 (48.61%)	100 (15.46%)	148 (22.87%)	188 (29.06%)	96 (14.84%)	124 (19.17%)	80 (12.36%)	135 (20.87%)	127 (19.63%)	33 (5.10%)
151–300	433 (32.53%)	58 (13.39%)	111 (25.64%)	88 (20.32%)	42 (9.70%)	59 (13.63%)	56 (12.93%)	80 (18.48%)	67 (15.47%)	16 (3.70%)
301–600	198 (14.88%)	22 (11.11%)	42 (21.21%)	31 (15.66%)	15 (7.58%)	34 (17.17%)	18 (9.09%)	33 (16.67%)	31 (15.66%)	4 (2.02%)
601–1,200	39 (2.93%)	4 (10.26%)	8 (20.51%)	7 (17.95%)	4 (10.26%)	4 (10.26%)	4 (10.26%)	7 (17.95%)	4 (10.26%)	0 (0%)
> 1,200	14 (1.06%)	0 (0%)	2 (14.29%)	3 (21.43%)	1 (7.14%)	2 (14.29%)	2 (14.29%)	1 (7.14%)	0 (0%)	0 (0%)
Chronic diseases[b] (*n* = 1,331)										
No	829 (62.28%)	94 (11.34%)	182 (21.95%)	160 (19.30%)	78 (9.41%)	118 (14.23%)	88 (10.62%)	145 (17.49%)	108 (13.03%)	21 (2.53%)
Yes	502 (37.72%)	89 (17.74%)	127 (25.30%)	159 (31.67%)	82 (16.33%)	105 (20.92%)	70 (13.94%)	111 (22.11%)	120 (23.90%)	32 (6.37%)
Cancer	10 (0.75%)	2 (20.00%)	5 (50.00%)	3 (30.00%)	3 (30.00%)	3 (30.00%)	3 (30.00%)	1 (10.00%)	3 (30.00%)	2 (20.00%)
Chronic kidney disease	11 (0.83%)	3 (27.27%)	4 (36.36%)	4 (36.36%)	5 (45.45%)	5 (45.45%)	4 (36.36%)	4 (36.36%)	4 (36.36%)	1 (9.09%)
Coronary artery disease	14 (1.05%)	4 (28.57%)	4 (28.57%)	4 (28.57%)	4 (28.57%)	3 (21.43%)	3 (21.43%)	3 (21.43%)	5 (35.71%)	2 (14.29%)
Asthma	23 (1.73%)	6 (26.09%)	8 (34.78%)	7 (30.43%)	2 (8.70%)	7 (30.43%)	8 (34.78%)	7 (30.43%)	4 (17.39%)	2 (8.70%)
Diabetes Mellitus	109 (8.19%)	20 (18.35%)	28 (25.69%)	27 (24.77%)	21 (19.27%)	23 (21.10%)	14 (12.84%)	24 (22.02%)	28 (25.69%)	5 (4.59%)
Hypertension	254 (19.08%)	41 (16.14%)	58 (22.83%)	76 (29.92%)	37 (14.57%)	49 (19.29%)	28 (11.02%)	58 (22.83%)	53 (20.87%)	13 (5.12%)
Dyslipidemia	80 (6.01%)	19 (23.75%)	24 (30.00%)	32 (40.00%)	20 (25.00%)	18 (22.50%)	18 (22.50%)	21 (26.25%)	23 (28.75%)	6 (7.50%)
Migraine	10 (0.75%)	2 (20.00%)	1 (10.00%)	5 (50.00%)	1 (10.00%)	3 (30.00%)	1 (10.00%)	2 (20.00%)	4 (40.00%)	2 (20.00%)
Peptic ulcer disease	17 (1.28%)	1 (5.88%)	3 (17.65%)	6 (35.29%)	1 (5.88%)	3 (17.65%)	1 (5.88%)	3 (17.65%)	6 (35.29%)	1 (5.88%)
Thalassemia	13 (0.98%)	0 (0%)	1 (7.69%)	1 (7.69%)	1 (7.69%)	4 (30.77%)	0 (0%)	1 (7.69%)	3 (23.08%)	0 (0%)
Thyroid	30 (2.25%)	6 (20.00%)	5 (16.67%)	12 (40.00%)	3 (10.00%)	9 (30.00%)	3 (10.00%)	8 (26.67%)	6 (20.00%)	1 (3.33%)
Rheumatoid/Gout	25 (1.88%)	5 (20.00%)	7 (28.00%)	7 (28.00%)	5 (20.00%)	9 (36.00%)	2 (8.00%)	7 (28.00%)	6 (24.00%)	2 (8.00%)
Allergies	28 (2.10%)	5 (17.86%)	8 (28.57%)	9 (32.14%)	7 (25.00%)	4 (14.29%)	7 (25.00%)	8 (28.57%)	12 (42.86%)	2 (7.14%)
Other diseases	62 (4.66%)	15 (24.19%)	22 (35.48%)	23 (37.10%)	14 (22.58%)	12 (19.35%)	12 (19.35%)	13 (20.97%)	15 (24.19%)	6 (9.68%)

n, number of available observations; SD, standard deviation.

[a] 1 USD is approximately 33 Thai baht.

[b] Some participants had multiple diseases.
